# Bioaugmentation strategies for methane production from digestate-alkali pretreated biomass via anaerobic digestion

**DOI:** 10.1186/s40643-025-00990-6

**Published:** 2025-12-10

**Authors:** Guangyu Bai, Yajing Yin, Zhiqiang Zhang, Yan Li, Yiyun Liu, Ran Wang, Zhi-Qiang He, Chong Han, Rong Zhang, Jia-Qi Cui, Bing-Zhi Li, He Bai

**Affiliations:** 1 State Key Laboratory of Synthetic Biology, Tianjin University, Tianjin, China; 2 Frontiers Science Center for Synthetic Biology (Ministry of Education), Tianjin, China; 3 School of Synthetic Biology and Biomanufacturing, Tianjin University, Tianjin, China; 4 Tianjin Huakan Environmental Protection Technol Co., Ltd, Tianjin, 300170 China; 5 Tianjin Huakan Group Company Limited, Tianjin, 300170 China; 6 Tianjin Key Laboratory of Food Biotechnology, College of Biotechnology and Food Science, Tianjin University of Commerce, Tianjin, 300134 China

**Keywords:** Lignocellulosic biomass, Pretreatment, Anaerobic digestion, Bioaugmentation, Methane

## Abstract

Sustainable management of agricultural and livestock wastes remains a major challenge in promoting the circular economy. In this study, a bioaugmentation strategy was applied to enhance methane production during the anaerobic digestion of lignocellulosic biomass. Under optimized conditions-including total solids content, pretreatment type, and inoculum source-the biogas yield and methane content reached 425 mL and 3.30%, respectively. Microbial community analysis indicated that *Bacteroidota*, *Firmicutes*, and *Proteobacteria* were the dominant phyla during anaerobic digestion, while the relative abundance of *Methanocorpusculum* increased steadily throughout the process. The anaerobic digestion performance of corn stover was further improved through the combined application of biostimulation and bioaugmentation, resulting in a methane content of 5.37%. Moreover, exogenous inoculation with genetically modified *Trichoderma reesei* SEU-7 and the methanogen *Methanosarcina barkeri* increased the biogas yield and methane content to 970 mL and 8.95%, respectively. Overall, these results demonstrate that rational optimization of anaerobic digestion parameters and microbial consortia can substantially enhance biomass degradation and bioconversion efficiency.

**Graphical Abstract:**

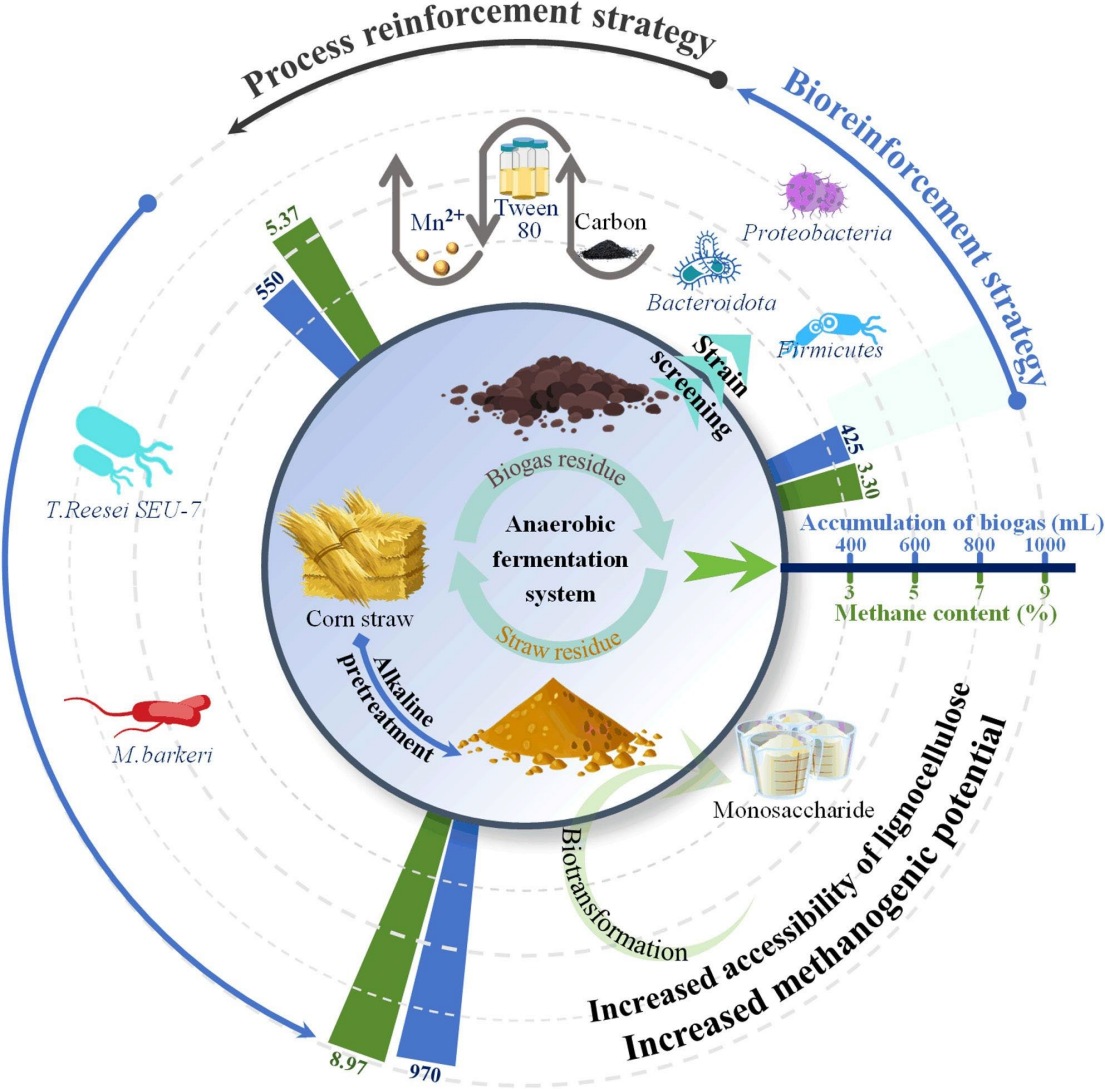

**Supplementary Information:**

The online version contains supplementary material available at 10.1186/s40643-025-00990-6.

## Introduction

China is a major agricultural producer, with rice, corn, and legumes serving as its primary grain crops. Harvesting these crops generates substantial amounts of residues, raising concerns regarding their disposal and reuse. Each year, China produces approximately 305 million tons of corn stover, of which about 214 million tons are available for collection and utilization (Sun et al. 2018). However, the overall utilization rate of corn stover in China remains below 80%, compared with rates exceeding 90% in many developed countries. Currently, corn stover is primarily used as fuel, fertilizer, or a raw material for building products (Choi et al. 2018). Nevertheless, seasonal surpluses have become increasingly common, resulting in large quantities of accumulated agricultural waste (Cantera et al. 2018). Traditionally, corn stover has been discarded outdoors or burned in open fields, practices that not only waste resources but also contribute to air pollution (Anshassi et al. 2022). Therefore, developing sustainable strategies for the utilization of agricultural residues is essential for environmental protection and resource recovery.

The primary resource utilization pathways for corn stover include its conversion into animal feed, bioenergy, and bio-based materials. Among these, anaerobic digestion and substrate preparation for edible mushroom cultivation are widely adopted. When used for biogas production, corn stover-derived biogas typically contains 55–70% methane, with an energy value of 20–25 MJ/m^3^ (Kumar et al. 2020). Meanwhile, China’s expanding livestock industry has led to a continuous increase in livestock and poultry production (Sana et al. 2023), accompanied by substantial growth in manure generation and associated environmental pressures. Livestock waste is rich in nitrogen, phosphorus, and potassium, offering considerable potential for nutrient recovery (Sana et al. 2021). Current manure management strategies primarily include energy recovery and fertilizer production, both of which are crucial for sustainable agricultural development. Among these strategies, anaerobic digestion plays a key role in converting livestock waste into biogas (Bao et al. 2023). Therefore, integrating the utilization of agricultural residues with livestock waste treatment to produce clean bioenergy represents a promising pathway for sustainable resource management (Rodriguez et al. 2022).

Anaerobic digestion, also known as anaerobic fermentation, is a multistage biological process driven by the synergistic activities of acidogenic bacteria, hydrogen-producing acetogens, and methanogenic archaea (Siddique et al., 2018). The widely accepted four-stage model comprises hydrolysis, acidogenesis, acetogenesis, and methanogenesis (Gaibor-Chávez et al. 2019). When corn stover is used as the substrate, cellulose and hemicellulose are hydrolyzed into soluble polysaccharides, whereas lignin shows high recalcitrance due to extensive intermolecular hydrogen bonding (Zhu et al. 2018). In recent years, various pretreatment methods, such as alkaline pretreatment, have been applied to enhance biomass digestibility by promoting polysaccharide solubilization and lignin removal (Cui et al. 2023). The hydrolyzed intermediates then undergo acidogenesis, acetogenesis, and methanogenesis, ultimately producing methane (Deng et al. 2017). Anaerobic digestion is a complex, enzyme-mediated microbial process, and fermentation temperature is considered a critical factor influencing lignocellulosic biomass conversion (Piercy et al. 2025). Although most anaerobic digestion systems currently operate under mesophilic or thermophilic conditions (Mukawa et al. 2024), limited research has focused on improving lignocellulosic digestion under uncontrolled or ambient temperatures. Additionally, the introduction of exogenous strains, including genetically engineered microorganisms and synthetic microbial consortia, can further enhance biomass degradation and methane production. Overall, anaerobic digestion performance is affected by multiple factors, including microbial community structure, substrate type, inoculum characteristics, and fermentation mode.

This study investigates the co-digestion of corn stover with livestock manure and biogas residues to address the challenges of stover accumulation and manure pollution. By optimizing total solids content, inoculum type, and fermentation conditions, this work aims to maximize methane production under non-controlled temperature conditions. Furthermore, bioaugmentation strategies-including the introduction of artificial microbial consortia, genetically engineered strains, and methanogenic archaea-were applied to enhance anaerobic digestion efficiency. High-throughput sequencing was also employed to monitor dynamic changes in microbial community structure throughout the fermentation process.

## Materials and methods

### Experimental materials

Corn stover used in this study was collected from suburban areas of Wuqing District, Tianjin, China. The stover was washed, air-dried, and ground, and particles between 20 and 80 mesh were selected for subsequent experiments. The moisture content was maintained below 10%, as determined using a moisture analyzer (Sartorius MA160-1CN, USA). Biogas residue, pig manure, and pig urine were obtained from local farms in Wuqing District. All chemicals used in this study were procured from Tianjin Yuanli Chemical Co., Ltd. (Tianjin, China). The elemental composition and content (wt%) of the raw materials are summarized in Table 1.

**Table 1:** The elemental composition and content of anaerobic digestion substrates (wt%)

	C	O	Cu	Cl	N	Si	Fe	Ca	Na	S
Corn stover	72.38	14.88	1.88	2.55	3.39	3.50	1.42	–	–	–
Pig manure	71.70	21.36	–	0.15	2.27	4.10	–	0.42	–	–
Pig urine	55.93	33.42	–	1.29	2.66	2.96	–	0.58	1.73	1.42

–, non-determined

### Experimental strains

The microbial strains used in this study included *Cronobacter* sp. (CGMCC 31759), *Bacteroides* sp. (CGMCC 31760), *Enterobacter* sp. (CGMCC 31757), and *Enterococcus* sp. (CGMCC 31758), which were isolated from biogas residues and preserved at the China General Microbiological Culture Collection Center (CGMCC). The parental strain *Trichoderma reesei* C30 and its genetically modified derivatives, *T. reesei* SEU-7 and *T. reesei* TRB1, were kindly provided by Southeast University. The methanogenic archaeon *Methanosarcina barkeri* was obtained from Nanjing Tech University.

### Construction and optimization of anaerobic digestion system

Raw and pretreated corn stover were used as substrates for anaerobic digestion, with pig manure or biogas residues serving as the inoculum and pig urine as the liquid medium. Fermentation was conducted in 500-mL blue-cap bottles equipped with gas outlets, and the resulting biogas was collected using gas bags. Biogas volume was recorded with a 2000-mL syringe, while methane content, expressed as a proportion of total biogas volume, was quantified using a high-precision methane detector (GT-903, China). Each reactor had a working volume of 300 mL and received a fixed inoculum dosage of 2% (v/v; 6 mL). The incubation temperature was maintained at 25 ± 2 °C. Corn stover inoculation ratios of 4%, 8%, 15%, 20%, and 30% (v/v) were applied over a 31-day anaerobic digestion period, during which biogas volume and methane content were monitored for 17 days. All experiments were performed in triplicate.

Corn stover was pretreated with either sodium hydroxide or ethylenediamine. Following pretreatment, the biomass was washed, air-dried, and subsequently used for anaerobic digestion. For sodium hydroxide pretreatment, 10 g of dry corn stover was mixed with 9 mL of sodium hydroxide solution (pH 13) in a sealed reaction vessel and heated in an oil bath at 130 °C for 30 min. For ethylenediamine pretreatment, 10 g of dry corn stover was mixed with 8 mL of ethylenediamine in a sealed vessel and heated in an oil bath at 130 °C for 60 min. After treatment, the vessels were cooled to room temperature under tap water, and the biomass was washed three times with 100 mL of distilled water, filtered through gauze, and dried at 60 °C until the moisture content was below 10%. The dried pretreated corn stover was stored in sealed bags at room temperature until use.

To enhance methane production, exogenous artificial microbial consortia (4 mL, OD₆₀₀ = 1.0), activated carbon, surfactants, and trace heavy metals were added at the start of anaerobic digestion. The dosages of activated carbon, surfactants, and trace heavy metals were optimized using a Box-Behnken response surface design in Design-Expert 8.0.6 software (Stat-Ease, Minneapolis, USA). In addition, *T. reesei* (2 mL, OD₆₀₀ = 1.0) and *M. barkeri* (2 mL, OD₆₀₀ = 1.0) were supplemented at the beginning of fermentation to promote anaerobic degradation.

### Detection of polysaccharide components in corn stover

For hydrolysis, 0.3 g of corn stover was mixed with 3 mL of 72% H₂SO₄ in a 100-mL blue-cap bottle and incubated at 30 °C on a shaker at 150 rpm for 60 min. Subsequently, 84 mL of distilled water was added, and the mixture was autoclaved at 121 °C for 60 min. The resulting hydrolysate was centrifuged at 12,000 rpm for 2 min, and the supernatant was filtered through a 0.22-μm aqueous membrane filter. Glucose and xylose concentrations were quantified using high-performance liquid chromatography (HPLC) equipped with an Aminex HPX-87H column (Bio-Rad, Hercules, CA) at 65 °C, with 5 mM H₂SO₄ as the mobile phase at a flow rate of 0.6 mL/min and detected using a Waters 2414 refractive index detector.

### Analysis of microbial community structure

To investigate microbial community dynamics during anaerobic digestion, samples were collected at the initial (day 8), middle (day 17), and final (day 31) stages of fermentation for community analysis. DNA was extracted using the cetyltrimethylammonium bromide (CTAB) method and verified by 1% agarose gel electrophoresis. Universal primers Arch344F (5′-ACGGGGYGCAGCAGGCGCGA-3′) and Arch915R (5′-GTGCTCCCCCGCCAATTCCT-3′) were used for polymerase chain reaction (PCR) amplification. PCR was conducted under the following conditions: initial denaturation at 95 °C for 3 min, followed by 32 cycles of 95 °C for 30 s, 55 °C for 30 s, and 72 °C for 45 s, with a final extension at 72 °C for 10 min. PCR products were pooled, visualized on 2% agarose gels, and subsequently purified. Sequencing was performed on the MiSeq PE300 platform (Novogene Bioinformatics Technology Co., Ltd., Beijing, China). Raw paired-end reads were merged using FLASH and demultiplexed using unique barcodes. High-quality reads were retained for downstream analysis. Operational taxonomic units (OTUs) were clustered at 97% sequence similarity using the RDP classifier with a Bayesian algorithm, and community composition was assessed at multiple taxonomic levels. OTU distribution was analyzed using USEARCH, while rarefaction curves, alpha-diversity indices, and Shannon–Wiener curves were generated using Mothur.

### Enzyme assay

The collected culture supernatant was appropriately diluted for enzyme assays. The activities of cellulolytic enzymes, including 1,4-*β*-D-glucosidase (pNPGase) and 1,4-*β*-D-glucan cellobiohydrolase (pNPCase), were measured according to previously reported protocols (Li et al. 2016, 2017).

### Data analysis

Experimental data were recorded and processed using Excel 2007, and significant differences between groups (*p* < 0.05) were determined by one-way analysis of variance (ANOVA). Mean comparisons were conducted using Duncan’s multiple range test in SPSS software (IBM Corp., Armonk, NY, USA).

## Results and discussion

### Characteristics of methane synthesis from biomass pretreated with biogas residue

#### Impact of biomass pretreatment on anaerobic digestion

**Fig. 1:**
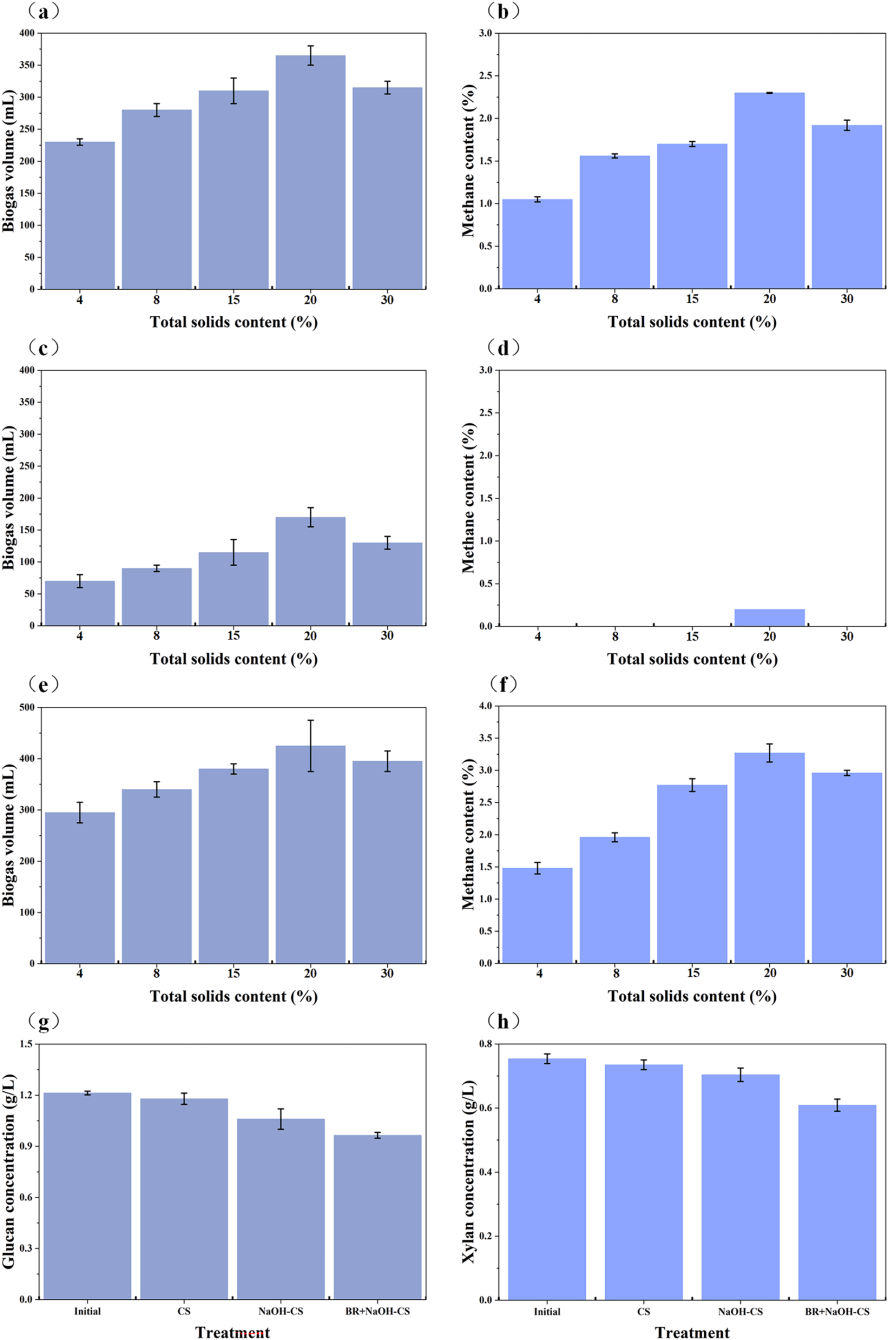
Anaerobic digestion characteristics and polysaccharide utilization of biomass under different conditions. **a** and **b** represents the anaerobic digestion characteristics of pig manure (PM) assisted with sodium hydroxide pretreated-corn stover (NaOH-CS); **c** and **d** represents the anaerobic digestion characteristics of PM assisted with ethylenediamine pretreated-corn stover (EDA-CS); **e** and **f** represents the anaerobic digestion characteristics of biogas residue (BR) assisted with NaOH-CS; **g** and **h** represent the concentration of glucan and xylan under anaerobic digestion conditions. Initial: the initial concentration of glucan and xylan in the non-treated CS. CS: PM assisted with CS; NaOH-CS: PM assisted with NaOH-CS; BR + NaOH-CS: biogas residue assisted with NaOH-CS

Lignocellulosic biomass is inherently recalcitrant, restricting the release and utilization of structural polysaccharides. To improve its biodegradability, various pretreatment strategies were applied to corn stover. Anaerobic digestion of the pretreated substrates revealed that sodium hydroxide pretreatment produced higher biogas yields and methane content than ethylenediamine pretreatment (Fig. 1). This result suggests that alkali-induced microstructural disruption enhances microbial and enzymatic degradation. In addition, the total solids content and carbon-to-nitrogen (C/N) ratio significantly influenced methane production during anaerobic digestion. As the total solids content of pretreated corn stover increased, biogas accumulation and methane concentration initially increased and then declined, indicating the presence of an optimal substrate loading for maximizing biomass degradation and methane production (Zhi et al. 2020). Specifically, when the total solids content of sodium hydroxide-pretreated corn stover reached 20% (C/N = 20:1), biogas yield and methane content peaked at 365 mL and 2.3%, respectively (Fig. 1). In contrast, ethylenediamine pretreatment resulted in lower biogas and methane production, likely due to amine-mediated alteration of the C/N ratio, which hindered anaerobic fermentation. Furthermore, polysaccharide recovery and lignin removal achieved by different pretreatment methods were also key determinants of biomass conversion efficiency.

#### Effect of inoculum on biomass anaerobic digestion

To enhance biomass degradation and methane production during anaerobic digestion, biogas residue was used as an inoculum to facilitate the fermentation of sodium hydroxide pretreated-corn stover (Fig. 1). As the proportion of sodium hydroxide pretreated-corn stover, biogas volume and methane content initially rose and then declined. Excessive biomass loading can impede mass transfer, thereby reducing methane production (Paritosh et al. 2021). Moreover, the addition of biogas residue positively correlated with biogas volume and methane content, emphasizing its role in enhancing gas synthesis and release. When sodium hydroxide pretreated-corn stover was applied at a 20% concentration, biogas volume and methane content peaked at 425 mL and 3.3%, respectively, surpassing the values observed when pig manure was used as the inoculum (Fig. 1). Biogas residue, derived from long-term fermentation of agricultural straw, contains microorganisms with strong lignocellulosic biomass degradation and conversion potential, thereby promoting methane accumulation (Peces et al. 2018). Furthermore, compared to pig manure, the microbial community in biogas residue demonstrated faster and more efficient biomass utilization without requiring prolonged adaptation or activation.

#### Effect of biogas residue-assisted anaerobic digestion on polysaccharide utilization

To evaluate polysaccharide utilization under different anaerobic digestion conditions, this study measured glucan and xylan concentrations in fermentation systems containing pig manure-corn stover, manure-sodium hydroxide pretreated-corn stover, and biogas residue-sodium hydroxide pretreated-corn stover, all at a C/N ratio of 20:1. As shown in Fig. 1, optimization of both substrate and inoculum types led to a gradual reduction in residual glucan and xylan in lignocellulosic biomass. Notably, anaerobic fermentation of biogas residue-assisted sodium hydroxide-pretreated corn stover achieved the highest glucan and xylan utilization. Alkali pretreatment roughened the biomass microstructure, facilitating polysaccharide release, while lignin removal further enhanced biomass degradation (Zhang et al. 2021). Furthermore, the microbial community in biogas residue substantially improved biomass degradation and conversion efficiency, thereby enhancing methane production during anaerobic digestion.

### Microbial community dynamics in biogas residue-assisted alkali pretreated-corn stover anaerobic digestion for methane production

Optimizing biomass type and inoculum selection in fermentation systems, combined with the use of biogas residue as an inoculum for alkali pretreated-corn stover, significantly enhances methane production. To elucidate the mechanisms underlying methane generation, microbial community analyses were performed at different fermentation stages of biogas residue-assisted sodium hydroxide pretreated-corn stover to identify key factors contributing to the improved methane yield.

#### Microbial community diversity and variability across fermentation stages

**Fig. 2:**
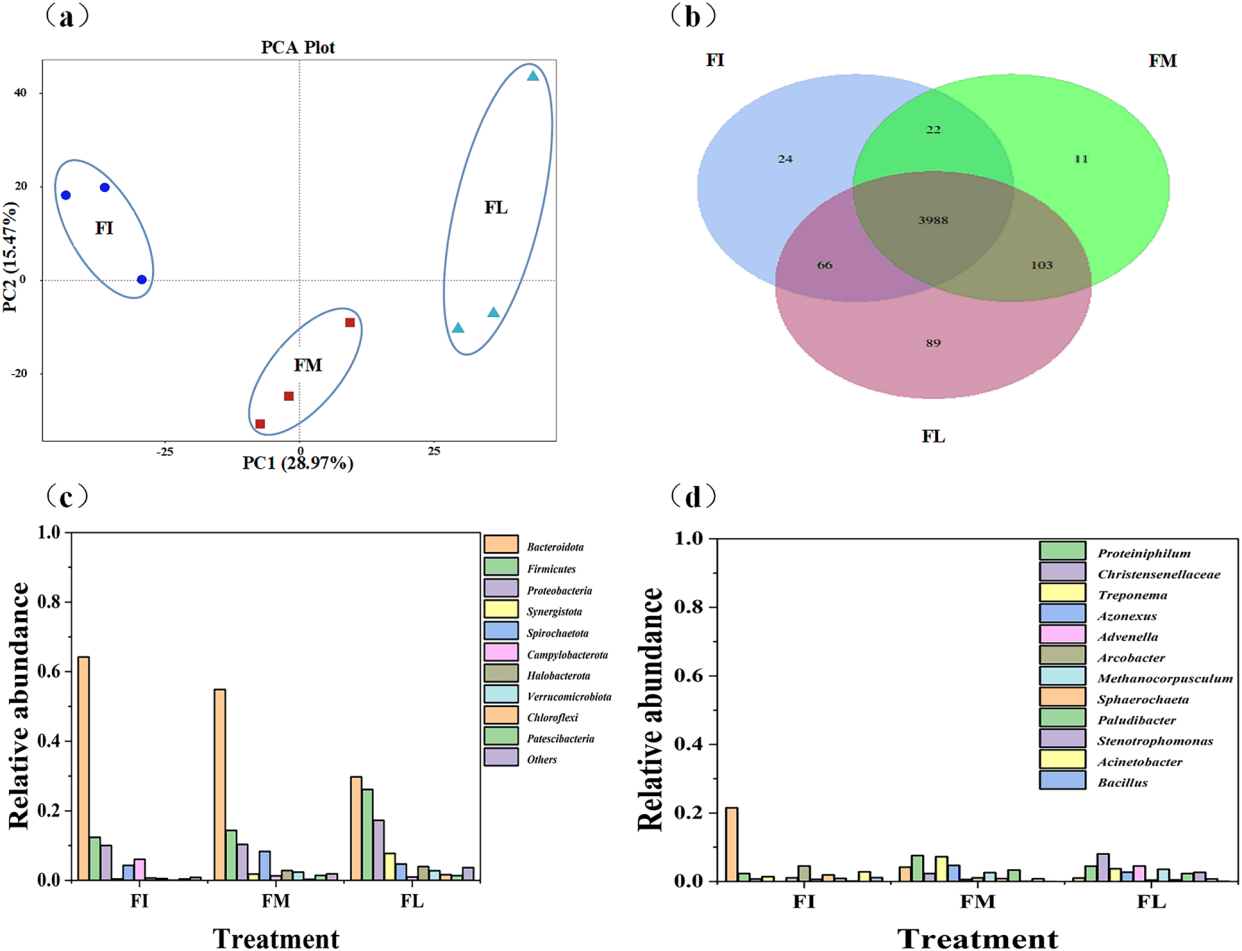
Composition of bacterial community structure during different stages of anaerobic digestion. **a** Principal component analysis of microbial community structure; **b** Venn diagram of microbial community structure; **c** Horizontal composition of phylum of microbial community structure; **d** Horizontal composition of microbial community structure; FI, early stage of fermentation; FI, mid-stage of fermentation; FI, late stage of fermentation

Principal component analysis revealed that the microbial community composition of biogas residue-assisted sodium hydroxide pretreated-corn stover varied across fermentation stages, as determined by high-throughput sequencing (Fig. 2a). The numbers of OTUs and diversity indices at each stage are presented in Table 2. OTU counts fluctuated throughout the fermentation process, generally increasing over time, with the highest count (1988) observed during the late fermentation stage, suggesting that methane production depends on synergistic interactions among diverse microorganisms to enhance substrate utilization and product conversion (Table 2). Furthermore, the Shannon and Simpson indices steadily increased, indicating the participation of a broad spectrum of functional microorganisms collaborating during anaerobic fermentation to produce methane (Pu et al. 2018). Cluster analysis of OTUs across fermentation stages identified approximately 4112 microbial species involved in the anaerobic fermentation process (Fig. 2b). Of these, 3988 species were shared across all stages, while 24, 11, and 89 species were uniquely present in the early, middle, and late stages, respectively. Microbial abundance peaked during the late fermentation stage and was lowest in the early stage.

**Table 2:** Bacterial community richness, evenness, and diversity values at different stages of biomass anaerobic digestion

Fermentation stage	OTUs	Chao1	Simpson	Shannon
FI	1474	1486	0.90	5.55
FM	1536	1558	0.92	6.05
FL	1988	2012	0.98	7.59

FI, initial stage of fermentation; FM, mid-stage of fermentation; FL, late stage of fermentation

#### Microbial community structure and dynamics in biogas residue-assisted alkali pretreated-corn stover anaerobic digestion

Microbial community analysis across different fermentation stages revealed that the phyla *Bacteroidota*, *Firmicutes*, *Proteobacteria*, *Synergistota*, and *Spirochaetota* played key roles in anaerobic digestion. Collectively, *Bacteroidota*, *Firmicutes*, and *Proteobacteria* accounted for over 70% of the total bacterial community, highlighting their central role in biomass degradation and conversion. Comparison of community composition across fermentation stages showed that the relative abundance of *Bacteroidota* decreased markedly in the late stage, declining from 64 to 30% (Fig. 2c). In contrast, *Firmicutes* and *Proteobacteria* steadily increased, reaching 26% and 17%, respectively, indicating that the microbial community composition dynamically changed during anaerobic digestion.

At the genus level, key taxa associated with anaerobic digestion included *Proteiniphilum*, *Treponema*, *Bacteroides*, *Christensenellaceae*, and *Methanocorpusculum* (Fig. 2d). During the early stage, *Arcobacter*, *Acinetobacter*, and *Sphaerochaeta* dominated, whereas *Paludibacter* and *Rikenellaceae* were more abundant in the mid-stage. In the late stage, *Advenella* and *Stenotrophomonas* emerged as dominant genera. As shown in Fig. 2d, the microbial community structure in the early stage differed substantially from that in the mid- and late stages. Early fermentation was characterized by *Bacteroides* (22% relative abundance), followed by *Arcobacter* and *Acinetobacter*. Members of *Bacteroidota*, known for their strong ability to degrade carbohydrates and proteins, contributed significantly to polysaccharide conversion and biomass breakdown (Zhu et al. 2019). As fermentation progressed, the relative abundances of *Bacteroides*, *Arcobacter*, and *Acinetobacter* gradually decreased (Fig. 2d), indicating a shift in anaerobic digestion from biomass degradation to methane production. Conversely, in the late stage, *Christensenellaceae*, *Advenella*, and *Proteiniphilum* increased. The relative abundance of *Methanocorpusculum* also rose progressively during fermentation (Fig. 2d), suggesting that methane-producing microorganisms increasingly contribute to biomass degradation and biogas accumulation over time. However, because *Methanocorpusculum* remained relatively scarce, strategies to enhance its population could further improve methane synthesis efficiency.

### Construction and optimization of an artificial mixed microbial consortium for methane synthesis via anaerobic digestion

#### Construction of the artificial mixed microbial consortium

In this study, four bacterial strains-*Cronobacter* sp., *Bacteroides* sp., *Enterobacter* sp., and *Enterococcus* sp.-were isolated from different stages of anaerobic digestion in a sodium hydroxide pretreated-corn stover system. A bioaugmentation strategy was employed to enhance methane production. However, when two randomly selected strains were introduced, cumulative biogas production was significantly lower than that of the control group, suggesting that the addition of exogenous strains intensified substrate competition and disrupted the balance of the indigenous microbial community (Brown et al. 2021). Similarly, fermentation assisted by an artificial mixed microbial consortium did not improve methane yields. Nevertheless, the combinations *Cronobacter* sp. + *Bacteroides* sp. and *Enterobacter* sp. + *Enterococcus* sp. achieved methane synthesis efficiencies comparable to the control group (Table S1). Although these combinations exhibited lower cumulative biogas production, their methane conversion efficiencies were significantly higher than those of other treatments, indicating that bioaugmentation can enhance methane yield relative to total biogas volume.

Increasing microbial cell density revealed that the combination *Cronobacter* sp. + *Bacteroides* sp. + *Enterococcus* sp. improved cumulative biogas production but did not increase methane yield (Table S1). When all four strains were introduced, cumulative biogas production reached 430 mL-similar to the control-while methane content increased to 4.30%. Given their documented biomass-degradation capabilities, these strains likely facilitated polysaccharide exposure and conversion within lignocellulose, thereby promoting substrate acidification and hydrogen production (Zhao et al. 2017). In contrast, the remaining artificial consortia exhibited lower cumulative biogas production and methane content compared with the control, highlighting the importance of selecting a compatible and stable bioaugmentation strategy to ensure efficient biomass degradation and conversion.

#### Optimization of the anaerobic digestion process

Although the artificial mixed microbial consortium-assisted biomass fermentation system enhanced methane synthesis potential, the anaerobic digestion process remained highly sensitive to fermentation conditions. Therefore, the effects of activated carbon concentration (A), Tween-80 content (B), and trace metal (Mn^2+^) concentration (C) were evaluated and optimized using response surface methodology, with methane content as the response variable (Table S2). The validity and significance of the regression model were assessed via analysis of variance (ANOVA) (Table S3). As shown in Table S3, the model was highly significant (*p* < 0.01), while the lack-of-fit term was insignificant (*p* > 0.05), indicating a strong fit with minimal influence from uncontrolled factors. The coefficients of determination (R^2^ = 0.99, adjusted R^2^ = 0.97) suggested that 97% of the variation in methane content could be explained by the model, confirming its predictive reliability. Among the quadratic terms, A^2^, B^2^, and C^2^ significantly affected methane content (*p* < 0.01), with the order of influence being Mn^2+^ > activated carbon > Tween-80. The quadratic polynomial regression model (Eq. 1) derived from this optimization is expressed as follows:

1 $$ \begin{aligned} {\text{Y}} = & \;{5}.{19} - 0.0{\text{21 A}} + \, 0.0{\text{16 B}} + 0.0{\text{32 C}}{-}0.0{7}0{\text{ AB}} \\ & + 0.0{\text{96 AC}}{-}0.{\text{13 BC}}{-}0.{\text{75 A}}^{{2}} {-}0.{\text{52 B}}^{{2}} - {1}.{\text{59 C}}^{{2}} \\ \end{aligned} $$

**Fig. 3:**
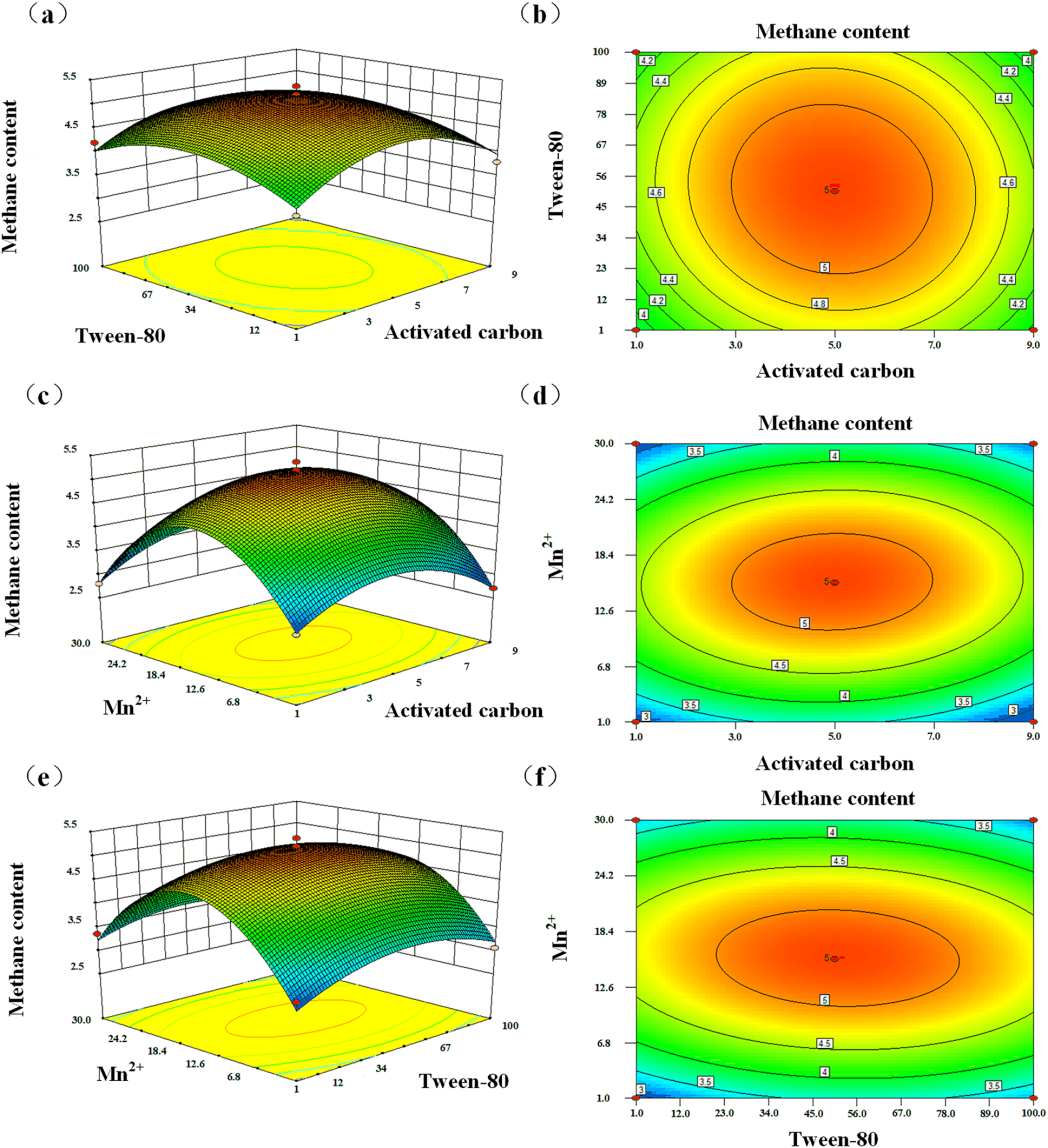
The effect of different exogenous additives on anaerobic digestion. **a** and **b** represent the response surface and contour lines of Tween-80 and activated carbon to methane content, respectively; **c** and **d** represent the response surface and contour lines of Mn^2+^ and activated carbon to methane content, respectively; **e** and **f** represent the response surface and contour lines of Mn^2+^ and Tween-80 to methane content, respectively

Following optimization, methane content increased to 5.37% under conditions of 5 g/L activated carbon, 50 mg/L Tween-80, and 15 mg/L Mn^2+^. Figure 3 illustrates the interaction effects of these factors on methane production during anaerobic digestion. When either activated carbon or Tween-80 concentration was held constant, methane content initially increased with the addition of the other factor but declined beyond a threshold. Moderate levels of activated carbon and surfactant sustained microbial activity and enhanced solvent interactions, thereby promoting biomass degradation and conversion (Evans et al. 2019). However, excessive concentrations exerted inhibitory or toxic effects on microbial growth and metabolism. The response surface generated by the regression model was relatively smooth, with nearly circular contour lines, indicating that the interaction between activated carbon and Tween-80 was not significant. In contrast, interactions between activated carbon and Mn^2+^ or Tween-80 and Mn^2+^ exhibited pronounced peaks followed by declines, reflected in steeper surfaces and elliptical contour lines. These results indicate that the interactions between activated carbon and Mn^2+^, as well as Tween-80 and Mn^2+^, were stronger than that between activated carbon and Tween-80. Previous studies have shown that trace metals enhance microbial activity and enzyme function in anaerobic digestion, thereby promoting lignocellulosic biomass degradation (Li et al. 2019). Specifically, Mn^2+^ acts as a cofactor for pyruvate carboxylase, arginase, and methyltransferases, playing a vital role in microbial metabolism. As shown in Table S3 and Fig. 3, the strongest interaction occurred between Tween-80 and Mn^2+^, followed by activated carbon and Mn^2+^, whereas the weakest interaction was between activated carbon and Tween-80 (Fig. 3).

### Enhancement strategies for anaerobic digestion of sodium hydroxide pretreated-corn stover

#### Methane production potential of different *T. reesei* strains in biomass fermentation

**Fig. 4:**
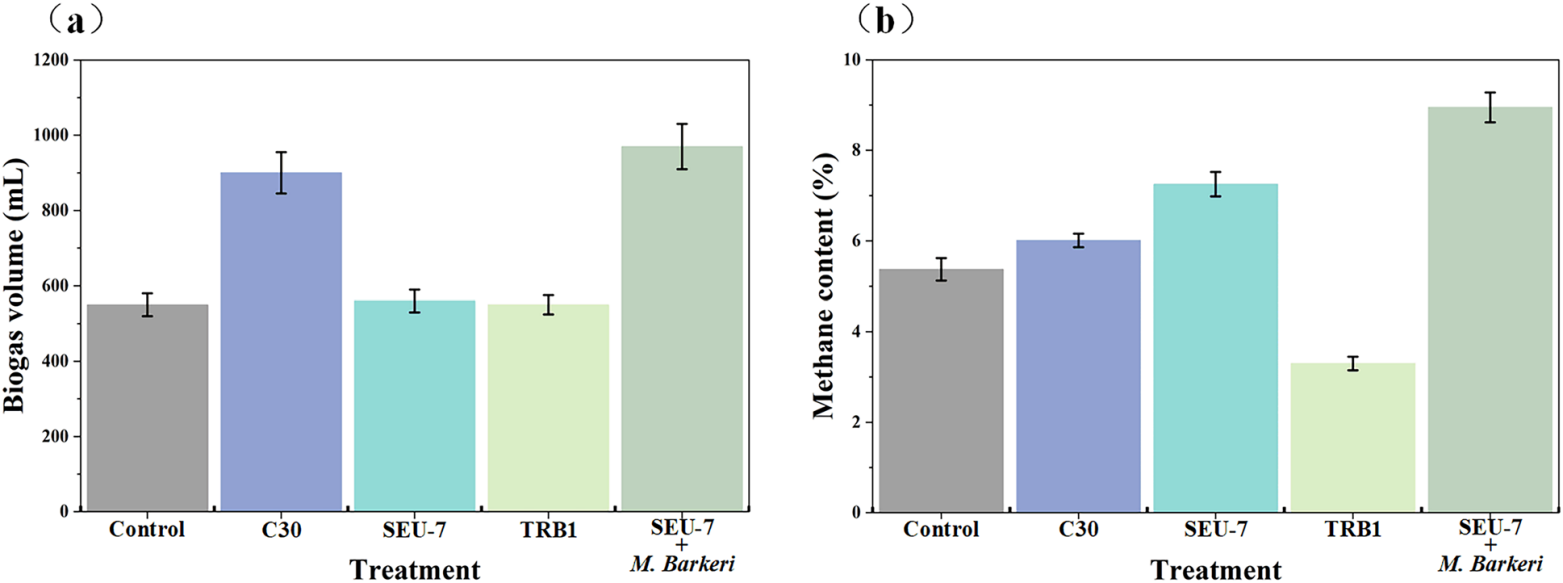
Biogas volume and methane content in bioaugmentation strategies assisted with anaerobic digestion. **a** biogas volume; **b** methane content. Control: non-added *T. reesei* or *M. barkeri* strains; C30: *T. reesei* C30 assisted with biomass anaerobic digestion; SEU-7: *T. reesei* SEU-7 assisted with biomass anaerobic digestion; TRB1: *T. reesei* TRB1 assisted with biomass anaerobic digestion; SEU-7 + *M. barkeri*: both *T. reesei* SEU-7 and *M. barkeri* assisted with biomass anaerobic digestion

To enhance the degradation efficiency of lignocellulosic biomass, this study introduced the exogenous strain *T. reesei* into an anaerobic digestion system for methane production. Incorporation of the wild-type strain *T. reesei* C30 into an artificial mixed microbial consortium within a sodium hydroxide pretreated-corn stover fermentation system significantly increased biogas production, reaching 870 mL, and elevated methane content to 6.01% (Fig. 4). Owing to its strong cellulolytic activity, *T. reesei* promoted glucan release, thereby facilitating downstream carbon metabolism (Li et al. 2017). To further enhance enzymatic activity, the *bgl1* (cel3a) gene was introduced into *T. reesei* C30 via *Agrobacterium*-mediated transformation, generating the genetically modified strain *T. reesei* SEU-7. Under cellulose or lactose induction, SEU-7 exhibited markedly higher cellulase and hemicellulase activities (Li et al. 2016).

As shown in Fig. 4, introduction of *T. reesei* SEU-7 into the artificial mixed microbial consortium-assisted fermentation system did not substantially increase cumulative biogas production; however, it raised methane content to 7.25%. This improvement is attributed to the strain’s high protein secretion capacity and inducible cellulase activity (Table S4), which enhanced biomass degradation and conversion. In contrast, introduction of *T. reesei* TRB1 into the anaerobic digestion system failed to improve either biogas accumulation or methane production, likely due to growth inhibition or reduced *β*-glucosidase activity caused by surfactant or manganese ion supplementation.

#### Methanogenic bacteria-assisted anaerobic digestion of biomass

Microbial community analysis of the sodium hydroxide pretreated-corn stover fermentation system revealed a relatively low abundance of methanogens, which limited biogas conversion and methane accumulation. To address this limitation, the exogenous methanogen *M. barkeri* was introduced to enhance methane production. As shown in Fig. 4, compared with the artificial mixed microbial consortium-assisted fermentation system containing *T. reesei* SEU-7, the addition of *M. barkeri* significantly increased both biogas yield and methane production. *M. barkeri* can utilize multiple metabolic pathways-including acetoclastic, hydrogenotrophic, and methylotrophic routes-making it a key methanogen in anaerobic digestion (Lyu et al., 2019). Moreover, relative to the system with *T. reesei* SEU-7, introduction of *M. barkeri* markedly enhanced cumulative biogas production (970 mL) and methane content (8.95%) (Fig. 4). Owing to its tolerance of environmental stressors such as high salinity and temperature fluctuations, *M. barkeri* effectively promoted biomass conversion. Nevertheless, although lignocellulosic biomass utilization was improved through bioaugmentation and biostimulation strategies during anaerobic digestion, several factors-including fermentation temperature and scale, cost-effectiveness, and operational feasibility-still require optimization to meet industrial application requirements.

### Mechanisms underlying the enhancement of anaerobic digestion via bioaugmentation and biostimulation strategies

**Fig. 5:**
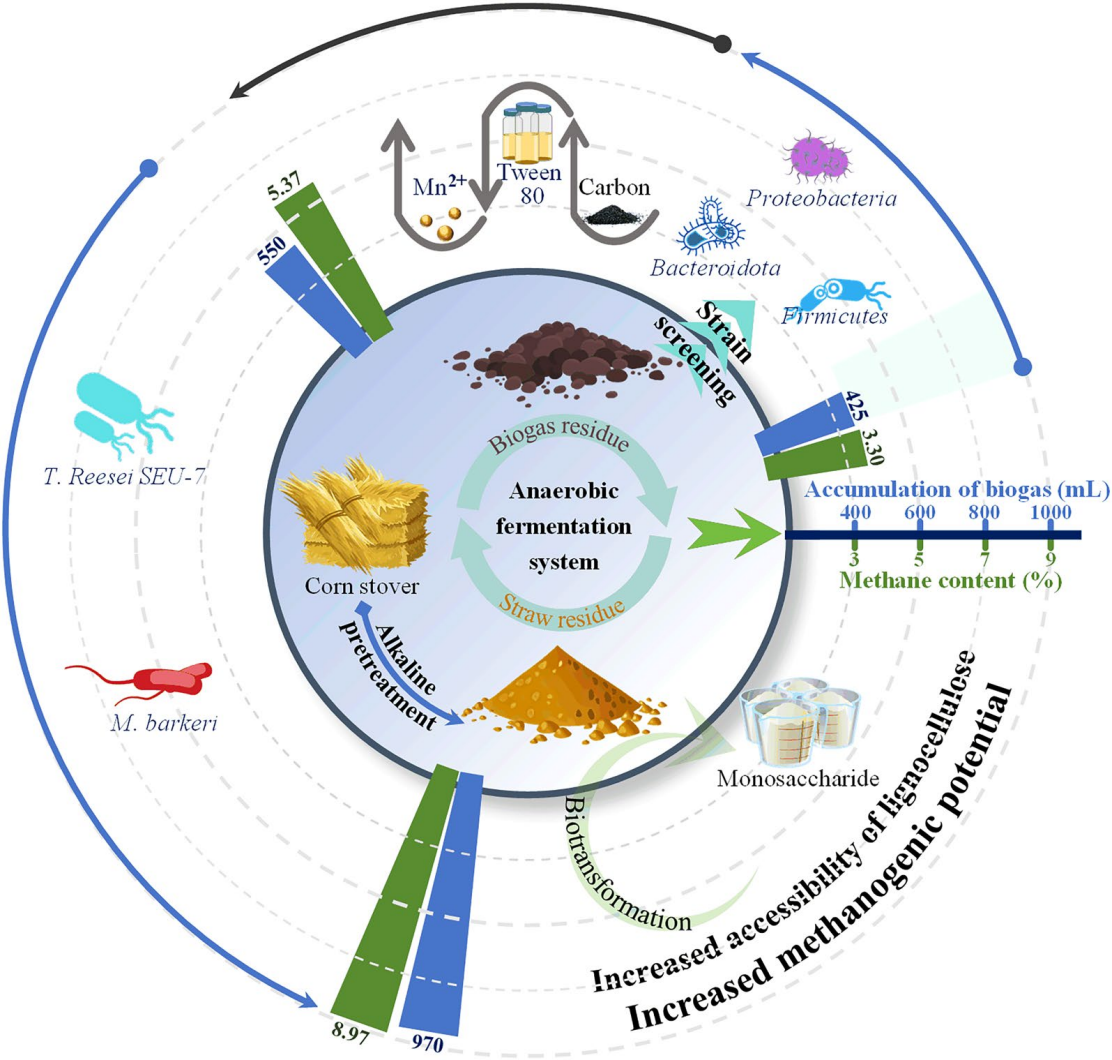
Process enhancement strategy assisted with anaerobic digestion for lignocellulosic biomass bioconversion

Despite its renewable nature, lignocellulosic biomass exhibits inherent recalcitrance, which severely limits its bioavailability in biorefining. In this study, sodium hydroxide pretreatment was employed to fractionate lignocellulosic components, thereby enhancing glucan and xylan utilization in the fermentation substrate (Fig. 5). Additionally, supplementation with long-term acclimated inoculum (biogas residual) improved anaerobic digestion performance, resulting in increased biogas yield and methane content. Microbial community analysis of the sodium hydroxide pretreated-corn stover system revealed that key biomass-degrading and metabolizing taxa, including *Bacteroidota*, *Firmicutes*, and *Proteobacteria*, played essential roles in anaerobic fermentation. Moreover, the relative abundance of the methanogen *Methanocorpusculum* increased throughout the fermentation process, promoting methane accumulation. Construction of an artificial mixed microbial consortium, combined with optimized fermentation parameters, achieved a biogas yield of 550 mL and a methane content of 5.37%. Furthermore, bioaugmentation with *T. reesei* SEU-7 (high lignocellulose degradation capacity) and *M. barkeri* (efficient methane conversion) further increased biogas accumulation to 970 mL and methane content to 8.95%.

## Conclusion

This study investigated bioaugmentation and biostimulation strategies to enhance methane production during the anaerobic digestion of lignocellulosic biomass. In the fermentation of sodium hydroxide-pretreated corn stover with digestate (C/N = 20:1), methane content reached 3.30%, with *Bacteroidota*, *Firmicutes*, and *Proteobacteria* identified as key contributors to the process. Moreover, the relative abundance of *Methanocorpusculum* increased as fermentation progressed. Introduction of an exogenous artificial microbial consortium-comprising *Cronobacter* sp., *Bacteroides* sp., *Enterobacter* sp., and *Enterococcus* sp.-together with optimized fermentation parameters (5 g/L activated carbon, 50 mg/L Tween-80, and 15 mg/L Mn^2+^) further enhanced biomass conversion, resulting in a methane content of 5.37% and cumulative biogas production of 550 mL. Furthermore, incorporation of *T. reesei* SEU-7 and *M. barkeri* into the anaerobic digestion system markedly improved performance, achieving a biogas yield of 970 mL and methane content of 8.95%.

## Supplementary Information

Supplementary Material 1

## Data Availability

All data analyzed during this study are included in this article.
