# Supplementary material for: Bioaugmentation strategies for methane production from digestate-alkali pretreated biomass via anaerobic digestion
Source: Bioresour Bioprocess. 2025 Dec 10;12(1):148. doi: 10.1186/s40643-025-00990-6 (PMC12696257; doi:10.1186/s40643-025-00990-6)
Supplement: Supplementary file 1 — Supplementary Material 1 [file 40643_2025_990_MOESM1_ESM.docx]

**Bioaugmentation strategies for** **methane production from digestate-alkali pretreated biomass via** **anaerobic digestion**

Guangyu Bai^d, #^, Yajing Yin^f, #^, Zhiqiang Zhang^e^, Yan Li^d^, Yiyun Liu^d^, Ran Wang ^a, b, c^, Zhi-Qiang He^a, b, c^, Chong Han^a, b, c^, Rong Zhang^d,^ *, Jia-Qi Cui^a, b, c,^ *, Bing-Zhi Li^a, b, c^, He Bai^d^.

^a^State Key Laboratory of Synthetic Biology, Tianjin University, Tianjin, China.

^b^Frontiers Science Center for Synthetic Biology (Ministry of Education).

^c^School of Synthetic Biology and Biomanufacturing, Tianjin University, Tianjin, China.

^d^Tianjin Huakan Environmental Protection Technol Co., Ltd, Tianjin 300170, China

^e^Tianjin Huakan Group Company Limited, Tianjin 300170, China

^f^Tianjin Key Laboratory of Food Biotechnology, College of Biotechnology and Food Science, Tianjin University of Commerce, Tianjin 300134, China

^#^These authors contributed equally to this work

^*^Corresponding authors:

Rong Zhang (E-mail): 395092626@qq.com

Jia-Qi Cui (E-mail): jqcui@tju.edu.cn

**Table S1. Biogas volume and methane content in anaerobic digestion assisted by artificial mixed microbial consortium**

| Artificial mixed microbial consortium | Biogas volume  (mL) | Methane content  (%) |
| --- | --- | --- |
| Control | 440 | 3.53 |
| *Cronobacter* sp. + *Bacteroides* sp. | 170 | 3.25 |
| *Cronobacter* sp. + *Enterobacter* sp. | 50 | 0.23 |
| *Cronobacter* sp. + *Enterococcus* sp. | 20 | 0.27 |
| *Bacteroides* sp. + *Enterobacter* sp. | 150 | 0.75 |
| *Bacteroides* sp. + *Enterococcus* sp. | 270 | 0.24 |
| *Enterobacter* sp. + *Enterococcus* sp. | 210 | 3.3 |
| *Cronobacter* sp.+ *Bacteroides* sp. + *Enterobacter* sp. | 270 | 1.05 |
| *Cronobacter* sp. + *Bacteroides* sp. + *Enterococcus* sp. | 300 | 1.75 |
| *Bacteroides* sp.+ *Enterobacter* sp. + *Enterococcus* sp. | 80 | 0.22 |
| *Cronobacter* sp. + *Bacteroides* sp. + *Enterobacter* sp. + *Enterococcus* sp. | 430 | 4.30 |

**Table S2. Box-Behnken test plan and results**

| Treatments | A: activated carbon  （g） | B: Tween-80  （mg/L） | C: Mn^2+^  （mg/L） | Methane content  (%) |
| --- | --- | --- | --- | --- |
| 1 | 1 | 1 | 15 | 3.73 |
| 2 | 9 | 1 | 15 | 3.79 |
| 3 | 1 | 100 | 15 | 4.2 |
| 4 | 9 | 100 | 15 | 3.98 |
| 5 | 1 | 50 | 1 | 2.9 |
| 6 | 9 | 50 | 1 | 2.7 |
| 7 | 1 | 50 | 30 | 2.8 |
| 8 | 9 | 50 | 30 | 2.98 |
| 9 | 5 | 1 | 1 | 3.06 |
| 10 | 5 | 100 | 1 | 3.06 |
| 11 | 5 | 1 | 30 | 3.37 |
| 12 | 4 | 100 | 30 | 2.83 |
| 13 | 5 | 50 | 15 | 5.13 |
| 14 | 5 | 50 | 15 | 5.37 |
| 15 | 5 | 50 | 15 | 5.15 |
| 16 | 5 | 50 | 15 | 5.12 |
| 17 | 5 | 50 | 15 | 5.20 |

**Table S3. Regression model and analysis of variance**

| Variation Source | Squares sum | Freedom | Mean square | F-values | *p*-values | Sig. |
| --- | --- | --- | --- | --- | --- | --- |
| Model | 15.56 | 9 | 1.73 | 53.85 | <0.0001 | * |
| A-activated carbon | 3.7 × 10^-3^ | 1 | 3.7 × 10^-3^ | 0.11 | 0.75 |  |
| B-Tween-80 | 2.1 × 10^-3^ | 1 | 2.1 × 10^-3^ | 0.07 | 0.80 |  |
| C-Mn^2+^ | 8.4 × 10^-3^ | 1 | 8.4 × 10^-3^ | 0.26 | 0.62 |  |
| AB | 0.02 | 1 | 0.02 | 0.62 | 0.46 |  |
| AC | 0.04 | 1 | 0.04 | 1.14 | 0.32 |  |
| BC | 0.07 | 1 | 0.07 | 2.21 | 0.18 |  |
| A^2^ | 2.37 | 1 | 2.37 | 73.92 | <0.0001 | * |
| B^2^ | 1.12 | 1 | 1.12 | 34.87 | 0.0006 | * |
| C^2^ | 10.71 | 1 | 10.71 | 333.57 | <0.0001 | * |
| Residual | 0.22 | 7 | 0.03 |  |  |  |
| Omission item | 0.18 | 3 | 0.06 | 5.83 | 0.06 |  |
| Net error | 0.04 | 4 | 0.01 |  |  |  |
| Sum | 15.79 | 16 |  |  |  |  |

Note: * represents significant differences (*p*<0.01).

**Table S4. Cellulolytic enzyme activities in the culture supernatant of different *T. reesei* strains assisted with anaerobic digestion system**

| Anaerobic digestion system | pNPGase  (IU/mL) | pNPCase  (IU/mL) |
| --- | --- | --- |
| Control | 96.2 ± 8.6 | 13.7 ± 2.2 |
| *T. reesei* C30 | 120.5 ± 17.3 | 14.8 ± 1.9 |
| *T. reesei* SEU-7 | 143.2 ± 21.5 | 17.1 ± 3.1 |
| *T. reesei* TRB1 | 128.3 ± 14.6 | 15.4 ± 2.3 |

pNPGase: 1, 4-*β*-D-glucosidase); encase :1, 4-*β*-D-glucan cellobiohydrolases
